# Vitreous Inflammatory Cytokines and Chemokines, Not Altered After Preoperative Adjunctive Conbercept Injection, but Associated With Early Postoperative Macular Edema in Patients With Proliferative Diabetic Retinopathy

**DOI:** 10.3389/fphys.2022.846003

**Published:** 2022-03-03

**Authors:** Hongyan Sun, Wenjun Zou, Zhengyu Zhang, Darui Huang, Jinxiang Zhao, Bing Qin, Ping Xie, Aime Mugisha, Qinghuai Liu, Zizhong Hu

**Affiliations:** ^1^The Affiliated Suqian First People’s Hospital of Nanjing Medical University, Suqian, China; ^2^Department of Ophthalmology, The First Affiliated Hospital of Nanjing Medical University, Nanjing, China; ^3^Department of Ophthalmology, The Affiliated Wuxi No. 2 People’s Hospital of Nanjing Medical University, Wuxi, China; ^4^Department of Ophthalmology, The Affiliated Huaian No. 1 People’s Hospital of Nanjing Medical University, Huaian, China

**Keywords:** anti-VEGF, macular edema, proliferative diabetic retinopathy, cytokines, inflammation, chemokines

## Abstract

**Purpose:**

To investigate the influence of preoperative adjunctive anti-VEGF drug (Conbercept) on vitreous inflammatory cytokines and chemokines profiles and whether those cytokines were associated with early macular edema (ME) after surgery for patients with proliferative diabetic retinopathy (PDR).

**Methods:**

In this *post hoc* analysis of the CONCEPT clinical trial, subjects with PDR underwent vitrectomy were included and vitreous samples were collected at the start of vitrectomy. Levels of vitreous VEGF, 17 inflammatory cytokines, and 11 chemokines were measured using Luminex multiplex technology. Subjects were then divided into groups based on with (Pre-IV) or without (No-Pre-IV) preoperative intravitreous injection of Conbercept; with or without early ME after surgery.

**Results:**

There was no difference between Pre-IV (13/30) and No-Pre-IV (7/29) concerning the ratio of patients with early ME (*p* = 0.17). After preoperative intravitreous injection of Conbercept, VEGF level dramatically decreased (*p* = 0.001), TNF-α (*p* = 0.002), and IP-10 (*p* = 0.018) increased in Pre-IV group. In patients with early ME after surgery, however, a number of cytokines increased, including IL-1β (*p* = 0.008), IL-2 (*p* = 0.023), IL-4 (*p* = 0.030), IL-9 (*p* = 0.02), IL-10 (*p* = 0.002), IL-12 (*p* = 0.001), IL-13 (*p* = 0.031), IL-17A (*p* = 0.008), TNF-α (*p* = 0.012), CXCL9 (*p* = 0.023), G-CSF (*p* = 0.019), MCP-1 (*p* = 0.048), and RANTES (*p* = 0.016).

**Conclusion:**

We found the preoperative adjunctive Conbercept injection has limited influence on the levels of vitreous inflammatory cytokines and chemokines in PDR. The elevated levels of a series of cytokines might be associated with early inflammation after vitrectomy, which may lead to postoperative ME.

## Introduction

Proliferative diabetic retinopathy (PDR) is characterized by retinal angiogenesis, which causes persistent vitreous hemorrhage (VH) and fibrous-associated tractional retinal detachment (Cheung et al., 2010). Patients with those conditions are generally treated with pars plana vitrectomy (PPV) to remove vitreous opacity, relieve retinal traction, and perform pan-retinal photocoagulation (PRP).

In the pathogenesis of DR, vascular endothelial growth factor (VEGF) is the primary angiogenesis factor, and its high level has been indicated to be related to the surgical difficulty (Zhang et al., 2013). Thus, the preoperative use of anti-VEGF antibodies has been widely proposed, especially for those with severe PDR, in order to decrease the surgery complexity (Yang and Xu, 2016; Castillo et al., 2017; Hu et al., 2021).

In addition to angiogenesis, evidence has also been accumulating that inflammatory and immune processes play an important role, with several studies showing a significant alteration of inflammatory factors and chemokines in ocular fluids of patients with PDR (Yoshida et al., 2015; Nawaz et al., 2019; Pan et al., 2021). High levels of inflammatory cytokines, such as interleukin family members and monocyte chemotactic protein-1 (MCP-1) (Felfeli et al., 2019; Abraham et al., 2021), are also strongly associated with macular edema, which if existed or progressed after vitrectomy, will compromise the visual prognosis of PDR (Im et al., 2017). Therefore, injection of triamcinolone acetonide at the end of vitrectomy may contribute to the reduction of postoperative inflammation and macular swelling (Takamura et al., 2018).

We have previously showed that after adjunctive anti-VEGF injection (Conbercept) before PPV, there was a remarkable and rapid decrease of intraocular VEGF-A (Hu et al., 2021), which was in line with the morphological findings (Hu et al., 2019). However, with the sudden drop of VEGF-A level and the regression of angiogenesis, how inflammatory factors and chemokines response in the ocular microenvironment still remains unclear. To the best of our knowledge, previous studies usually measured the cytokines over weeks or months after IV-anti-VEGF treatment, and mainly for diabetic macular edema (DME) (Jeon and Lee, 2012; Hillier et al., 2018; Mastropasqua et al., 2018; Cacciamani et al., 2019; Felfeli et al., 2019; Wei et al., 2019; Juncal et al., 2020; Imazeki et al., 2021; Xavier et al., 2021), few focused on the early-stage changes of cytokines after IV-anti-VEGF. In addition, the changes of cytokines seemed controversial among studies, some of which reported the cytokines decreased (Cacciamani et al., 2019; Felfeli et al., 2019; Coughlin et al., 2020; Imazeki et al., 2021), while others found the opposite (Jeon and Lee, 2012; Mastropasqua et al., 2018; Xavier et al., 2021).

In the current study, we aimed to investigate whether the preoperative adjunctive injection of Conbercept changes the early-stage retinal inflammatory status. To determine this, we firstly compared the early ME proportion after surgery between patients with or without adjunctive injection of anti-VEGF drug. Secondly, we measured the levels of cytokines between the two treatment groups. Finally, we evaluated the levels of cytokines between patients with or without early ME.

## Materials and Methods

### Participants

This was a *post hoc* analysis of the CONCEPT clinical trial. The CONCEPT clinical trial was previously described (Hu et al., 2021) and the clinical trial was registered at https://clinicaltrials.gov/ (ID NCT03506750). This study adhered to the tenets of the Declaration of Helsinki, and was approved by Ethic Committee of First Affiliated Hospital of Nanjing Medical University (2017-SR-283). We obtained the informed written consent from each included patient prior to enrollment.

The CONCEPT clinical trial was performed from June 2017 to January 2018 at The First Affiliated Hospital of Nanjing Medical University. The main inclusion criteria were patients (1) at least 18 years old; (2) with PDR diagnosis and with necessity of PPV surgery; and (3) with high-image-quality optical coherence tomography (OCT) examination 1–4 weeks after PPV surgery. The main exclusion criteria were patients (1) with previous intraocular surgery history; (2) with other retinal diseases or neovascularization glaucoma; or (3) with failure to obtain qualified samples. A random number generator was used to allocate participants to groups receiving no intravitreous injection (No-pre-IV, *n* = 30), or injections of Conbercept 4–7 days (Pre-IV, *n* = 29) before PPV. Based on our pilot study of VEGF-A expression in patients with or without IV, to detect a difference of at least 100.0 ± 100.0 ng/ml VEGF-A between the two groups with 95% power and significance level (alpha) of 0.05, the minimum sample size we estimated was 25. In Pre-IV group, participants were administrated with 0.5 mg/0.05 mL Conbercept (Chengdu Kanghong Biotech, Inc., Chengdu, Sichuan, China) 4–7 days before surgery. We previously has demonstrated the non-diabetic idiopathic macular hole (iMH) and epiretinal membrane (iERM) can be combined as “healthy control” for vitreoretinal diseases (Chen et al., 2021), we herein included 20 eyes with iMH or iERM in Control group.

### Sample Collection

The vitreous samples for all patients were collected with a vitreous cutter at the start of vitrectomy before intraocular infusion. Approximately 0.5–0.8 mL samples were firstly harvested into sterile tubes, immediately placed on ice, centrifuged at 1,500 rpm for 5 min to remove the cells and debris, and then stored at −80°C until analyzed.

### Measurement of Vitreous Cytokines

**FIGURE 1:**
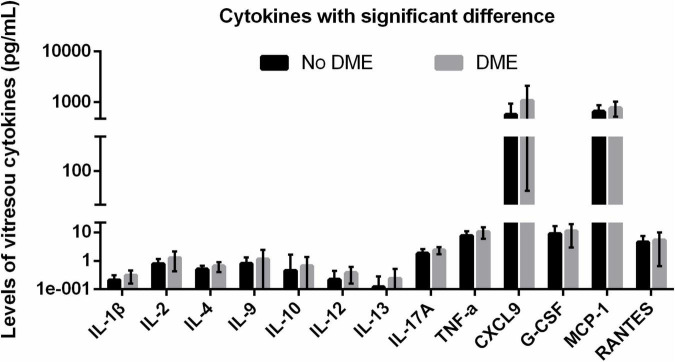
DME-related upregulated vitreous cytokines with statistical significance. DME, diabetic macular edema.

The cytokines we measured included VEGF, inflammatory cytokines (IL-1β, IL-1ra, IL-2, IL-4, IL-5, IL-6, IL-7, IL-8, IL-9, IL-10, IL-12, IL-13, L-15, IL-17A, IL-18, IFN-g, and TNF-a), and chemokines (CXCL9, CXCL12, MIF, G-CSF, GM-CSF, M-CSF, IP-10, MCP-1, MIP-1a, MIP-1b, and RANTES) (Figure 1). The concentrations of vitreous cytokines were measured using Luminex multiplex technology (Bio-Rad Laboratories, Hercules, CA, United States) according to the manufacturer’s protocol with the assistance of Wayen Biotechnologies (Shanghai, Inc.). Briefly, 50 μl of vitreous sample or provided standard was added to 96-well micro-titer plate. After incubation at room temperature, diluted biotin antibody was then used to incubate with the samples, then added Streptavidin-PE for further incubation followed by wash buffer. Finally, the plate was read on the Bio-Plex MAGPIX System (Bio-Rad Laboratories).

### Optical Coherence Tomography

Retinal sectional images of the macula were acquired using SD-OCT Cirrus (Carl Zeiss, Meditec, Germany). OCT was used to determine the presence or absence of ME, which manifests as diffuse retinal thickening, cystoid macular edema and serous retinal detachment. We determined the “early phase” as 1–4 weeks after PPV surgery. Patients with or without ME during follow-ups within the “early phase” period were divided to compare the vitreous levels of cytokines. We measured the retinal thickness at the central 1-mm subfield on the Early Treatment Diabetic Retinopathy Study (ETDRS) grid on the OCT maps constructed by raster scans.

### Statistical Analysis

All analyses were performed using SPSS 20.0 (SPSS, Inc., Chicago, IL, United States). Chi-square test was used to compare baseline differences in gender, proportion of types of PDR [vitreous hemorrhage (VH), or fibrovascular membrane (FVM), or combined], and number of patients with preoperative PRP. Independent *t*-test was used to compare baseline differences in age and level of hemoglobin A1C. For duration of vision loss, LogMAR BCVA, IOP, and duration of diabetes which did not pass the Kolmogorov–Smirnov normality test, the baseline differences of these parameters were compared using Kruskal–Wallis variance analysis.

For the cytokines, the variables were firstly checked for normality using Kolmogorov–Smirnov test. Non-parametric data were expressed as median and range and analyzed by Kruskal–Wallis variance analysis. Continuous parametric data were presented as means ± standard deviation of the mean, and were analyzed using a one-way analysis of variance (ANOVA) followed by a Bonferroni test. To decrease the possibility the type 1 errors, the adjusted *P*-value was divided by the number of comparisons (adjusted *P* = 0.05/n), and if a calculated *P*-value was smaller than the adjusted *P*-value, the difference was with statistical significance. Correlation test was performed using Pearson correlation test between the upregulated cytokines (e.g., IL-1β) and clinical characteristics (e.g., age).

## Results

### Preoperative Characteristics

A total of 30 patients with PDR were included in the No-Pre-IV group and 29 were in the Pre-IV group. The baseline characteristics of each group are showed in Table 1. The main interval between Pre-IV and PPV was 135.33 ± 27.57 h. There was no difference in age, gender, duration of vision loss and diabetes, ratio of PDR types, log MAR BCVA, IOP, hemoglobin A1c, and previous PRP history (each *p* > 0.05).

**TABLE 1:** Baseline characteristics of included patients with PDR.

		No-Pre-IV	Pre-IV	*P*-value
Number		30	29	–
Age (mean ± SD, yrs)		49.14 ± 10.58	52.57 ± 14.86	0.313^#^
Female gender, no. (%)		13 (43.3)	15 (51.7)	0.519^†^
Duration of vision loss (range, mths)		3.27 (0.17–12)	1.0 (0.33–38)	0.288*
PDR type	VH, no (%)	13	6	0.177^†^
	FVM, no (%)	6	8	
	FVM and VH, no (%)	11	15	
LogMAR BCVA		2.30 (0.30–2.60)	2.00 (0.30–2.60)	0.812*
IOP		12.4 (10.2 – 31.0)	14.5 (10.0 – 24.0)	0.222*
Duration of diabetes (range, yrs)		10.0 (1 – 30)	14 (1 – 50)	0.360*
Hemoglobin A1C (range)		7.0 ± 1.04	7.1 ± 0.99	0.688^#^
PRP history, no. (%)		9 (30.0)	8 (27.6)	0.838^†^
Early DME		13	7	0.17

*Kruskal–Wallis variance analysis (did not pass the Kolmogorov–Smirnov normality test).

*^†^Chi-square test.*

*^#^Independent t-test.*

*PDR, proliferative diabetic retinopathy; No-Pre-IV, PDR eyes with no preoperative injection of anti-VEGF drug; Pre-IV, PDR eyes with preoperative injection of anti-VEGF drug; VH, vitreous hemorrhage; FVM, fibrovascular membrane; BCVA, best corrected visual acuity; IOP, intraocular pressure; PRP, panretinal photocoagulation.*

### Levels of Cytokines After Pre-IV

The mean vitreous levels of most inflammatory cytokines were higher in PDR patients either in No-Pre-IV group or in Pre-IV group, as compared with those in control patients (Tables 2, 3).

**TABLE 2:** Vitreous levels of inflammatory cytokines among the groups of Control, No-Pre-IV, and Pre-IV.

	Control^#^	No-pre-IV	Pre-IV	*P*-value (no-pre-IV vs. ctrl)	*P*-value (pre-IV vs. ctrl)	*P*-value (no-pre-IV vs. pre-IV)
VEGF-A*	0 (0 – 0)	0 (0 – 1512.2)	0 (0–80)	0.001	1.00	0.001
	0.00 ± 0.00	158.14 ± 401.35	2.76 ± 14.88			
IL-1b*	0.07 (0.05–0.22)	0.18 (0.07–0.54)	0.24 (0.10–0.54)	<0.001	<0.001	0.803
	0.09 ± 0.04	0.21 ± 0.11	0.27 ± 0.15			
IL-1ra*	12.10 (8.38–72.73)	27.28 (12.10–666.96)	25.67 (12.10–808.94)	<0.001	<0.001	1.0
	15.42 ± 13.96	68.39 ± 131.51	61.21 ± 148.47			
IL-2*	0.23 (0.00–0.40)	0.61 (0.27–1.85)	0.98 (0.36–2.71)	<0.001	<0.001	0.18
	0.22 ± 0.11	0.74 ± 0.39	1.16 ± 0.72			
IL-4^†^	0.14 (0.10–0.39)	0.46 (0.27–1.05)	0.55 (0.31–1.05)	<0.001	<0.001	0.117
	0.15 ± 0.07	0.52 ± 0.19	0.60 ± 0.23			
IL-5*	0.00 (0.00–0.00)	0.00 (0.00–3.72)	0.41 (0.00–5.37)	0.078	<0.001	0.115
	0.00 ± 0.00	0.58 ± 1.11	1.05 ± 1.42			
IL-6*	0.00 (0.00–0.95)	2.44 (0.09–91.62)	7.25 (1.05–61.75)	<0.001	<0.001	0.081
	0.09 ± 0.22	8.86 ± 18.48	12.45 ± 14.05			
IL-7*	14.09 (6.02–25.63)	18.95 (4.28–50.55)	13.52 (3.47–68.82)	>0.05	>0.05	>0.05
	15.74 ± 5.66	20.65 ± 11.00	18.09 ± 13.84			
IL-8*	2.13 (1.18–52.43)	53.37 (12.69–609.25)	96.64 (18.20–1758.28)	<0.001	<0.001	0.032
	5.85 ± 11.93	78.21 ± 110.93	217.23 ± 327.88			
IL-9*	0.02 (0.00–0.62)	0.76 (0.00–2.02)	0.89 (0.11–3.76)	<0.001	<0.001	1.0
	0.17 ± 0.22	0.91 ± 0.54	1.02 ± 0.76			
IL-10*	0.00 (0.00–0.20)	0.20 (0.00–1.36)	0.32 (0.00–7.14)	<0.001	<0.001	1.0
	0.01 ± 0.04	0.31 ± 0.32	0.70 ± 1.39			
IL-12*	0.16 (0.00–0.62)	0.29 (0.00–0.62)	0.22 (0.00–0.73)	>0.05	>0.05	>0.05
	0.17 ± 0.14	0.28 ± 0.21	0.28 ± 0.24			
IL-13*	0.30 (0.00–1.01)	0.14 (0.00–1.07)	0.09 (0.00–0.82)	>0.05	>0.05	>0.05
	0.38 ± 0.36	0.17 ± 0.23	0.15 ± 0.19			
L-15*	0.00 (0.00–0.00)	0.00 (0.00–0.00)	0.00 (0.00–0.00)	>0.05	>0.05	>0.05
	0.00 ± 0.00	0.00 ± 0.00	0.00 ± 0.00			
IL-17A*	0.84 (0.64–1.24)	1.95 (0.74–3.97)	2.25 (0.74–3.36)	<0.001	<0.001	1.0
	0.82 ± 0.16	2.01 ± 0.76	2.06 ± 0.80			
IL-18^†^	59.75 (41.37–88.68)	76.52 (13.95–111.60)	88.68 (0.00–169.54)	0.076	0.001	0.053
	61.52 ± 14.67	75.16 ± 24.56	88.61 ± 32.72			
IFN-g*	7.01 (5.00–27.00)	32.83 (7.00–77.00)	43.40 (19.00–78.00)	<0.001	<0.001	0.139
	8.65 ± 4.98	38.20 ± 17.95	48.84 ± 16.85			
TNF-a^†^	1.49 (0.94–2.49)	7.21 (2.96–16.72)	8.78 (3.87–22.09)	<0.001	<0.001	**0.002**
	1.61 ± 0.50	7.30 ± 2.79	10.01 ± 4.49			

*Kruskal–Wallis variance analysis (did not pass the Kolmogorov–Smirnov normality test), p-value < 0.017 (0.05/3) was considered with statistical significance.

*^†^One-way ANOVA, Bonferroni adjustment, p-value < 0.017 (0.05/3) was considered with statistical significance.*

*^#^Patients with idiopathic macular hole or epiretinal membrane were included as controls.*

*No-Pre-IV, PDR eyes with no preoperative injection of anti-VEGF drug; Pre-IV, PDR eyes with preoperative injection of anti-VEGF drug; IL, interleukin; IFN-g, interferon-g; TNF-a, tumor necrosis factor-a. Bold values means with statistical significance.*

**TABLE 3:** Vitreous levels of chemokines among the groups of Control, No-Pre-IV, and Pre-IV.

	Control^#^	No-pre-IV	Pre-IV	*P*-value (no-pre-IV vs. ctrl)	*P*-value (pre-IV vs. ctrl)	*P*-value (no-pre-IV vs. pre-IV)
CXCL12^†^	560.52 (374.77–920.53)	658.72 (260.14–1329.40)	751.31 (222.29–1775.91)	0.031	0.005	0.454
	560.52 ± 146.23	743.90 ± 293.43	789.38 ± 334.87			
CXCL9*	100.00 (100.00–447.84)	496.48 (100.00–1536.56)	691.81 (132.07–4373.25)	<0.001	<0.001	0.052
	133.80 ± 92.60	520.77 ± 379.72	929.08 ± 856.47			
MIF*	6763.10 (2442.00–47716.34)	34816.58 (4798.81–165505.24)	34235.34 (7570.28–191557.87)	<0.001	<0.001	1.0
	10632.19 ± 10493.40	52724.10 ± 37570.80	56147.34 ± 45342.76			
G-CSF*	3.65 (3.26–4.86)	7.23 (3.65–26.19)	7.90 (4.05–45.54)	<0.001	<0.001	1.0
	3.76 ± 0.38	8.26 ± 4.32	10.92 ± 9.76			
GM-CSF*	0.00 (0.00–0.45)	0.00 (0.00–0.45)	0.00 (0.00–0.85)	0.487	0.01	0.23
	0.00 ± 0.00	0.04 ± 0.11	0.11 ± 0.21			
M-CSF*	126.26 (0.00–302.05)	82.19 (0.00–535.72)	90.03 (0.00–400.00)	>0.05	>0.05	>0.05
	141.35 ± 90.59	101.86 ± 117.50	124.53 ± 104.84			
IP-10*	947.17 (248.53–2753.13)	4479.02 (961.34–30513.23)	11128.20 (3889.47–56177.70)	<0.001	<0.001	**0.018**
	1031.32 ± 634.76	7210.32 ± 6534.27	15278.9914 ± 13939.86850			
MCP-1^†^	138.29 (90.93–247.80)	591.47 (120.57–1226.51)	733.12 (357.64–1146.69)	<0.001	<0.001	0.075
	145.49 ± 40.67	639.50 ± 243.77	733.09 ± 226.96			
MIP-1a*	0.28 (0.17–0.38)	1.23 (0.20–26.42)	2.05 (0.65–11.02)	<0.001	<0.001	0.249
	0.27 ± 0.05	3.13 ± 5.70	2.81 ± 2.69			
MIP-1b*	1.60 (0.80–5.89)	6.73 (1.62–68.61)	7.40 (2.72–94.61)	<0.001	<0.001	1.0
	1.92 ± 1.09	11.80 ± 16.18	14.11 ± 18.70			
RANTES*	1.60 (0.99–2.71)	4.24 (1.20–19.14)	4.24 (2.54–9.65)	<0.001	<0.001	1.0
	1.56 ± 0.36	4.98 ± 3.31	4.54 ± 1.82			

*Kruskal–Wallis variance analysis (did not pass the Kolmogorov–Smirnov normality test), p-value < 0.017 (0.05/3) was considered with statistical significance.

*^†^One-way ANOVA, Bonferroni adjustment, p-value < 0.017 (0.05/3) was considered with statistical significance.*

*^#^Patients with idiopathic macular hole or epiretinal membrane were included as controls.*

*No-Pre-IV, PDR eyes with no preoperative injection of anti-VEGF drug; Pre-IV, PDR eyes with preoperative injection of anti-VEGF drug; CXCL, C-X-C chemokine ligand; MIF, macrophage-inhibiting factor; G-CSF, granulocyte-colony stimulating factor; GM-CSF, granulocyte-macrophage colony stimulating factor; M-CSF, macrophage colony stimulating factor; IP-10, interferon-inducible protein-10; MCP-1, monocyte chemoattractant protein-1; MIP, macrophage inflammatory protein; RANTES (CCL2), regulated upon activation normal T cell expressed and secreted. Bold values means with statistical significance.*

After preoperative intravitreous injection of Conbercept (IVC), the VEGF level dramatically decreased (158.14 ± 401.35 pg/mL in No-Pre-IV group versus 2.76 ± 14.88 pg/mL in Pre-IV group, *p* = 0.001). With the drop of VEGF level, however, most of inflammatory cytokines and chemokines did not alter remarkably. The inflammatory cytokines and chemokines increased with or approaching statistical significance were IL-6 (from 8.86 ± 18.48 to 12.45 ± 14.05 pg/mL, *p* = 0.081), IL-8 (from 78.21 ± 110.93 to 217.23 ± 327.88 pg/mL, *p* = 0.032), IL-18 (from 75.16 ± 24.56 to 88.61 ± 32.72 pg/mL, *p* = 0.053), TNF-α (from 7.30 ± 2.79 to 10.01 ± 4.49 pg/mL, *p* = 0.002), CXCL9 (from 520.77 ± 379.72 to 929.08 ± 856.47 pg/mL, *p* = 0.052), IP-10 (from 7210.32 ± 6534.27 to 15278.9914 ± 13939.86850 pg/mL, *p* = 0.018), and MCP-1 (from 639.50 ± 243.77 to 733.09 ± 226.96 pg/mL, *p* = 0.075) (Tables 2, 3).

### Levels of Cytokines in Patients With Early Macular Edema

During the early phase (1–4 weeks) follow-up, 13 out of 30 patients in Pre-IV group and 7 out of 29 patients in Pre-IV group were detected with ME on OCT images (*p* = 0.17) (Table 1). There was also no difference between the main central retinal thickness (*p* > 0.05).

Next, we retrospectively divided the PDR patients into two groups based on with or without early ME. In patients with early ME, a number of inflammatory cytokines increased, including IL-1β (*p* = 0.008), IL-2 (*p* = 0.023), IL-4 (*p* = 0.030), IL-9 (*p* = 0.02), IL-10 (*p* = 0.002), IL-12 (*p* = 0.001), IL-13 (*p* = 0.031), IL-17A (*p* = 0.008), and TNF-α (*p* = 0.012). In addition, several chemokines also increased in patients with early ME, such as CXCL9 (*p* = 0.023), G-CSF (*p* = 0.019), MCP-1 (*p* = 0.048), and RANTES (*p* = 0.016) (Table 4). These results indicated that the elevated levels of a series of cytokines at the time of surgery might be associated with early postoperative ME.

**TABLE 4:** Inflammatory cytokines and chemokines between PDR patients with or with no early ME after surgery.

		No DME	DME	*p*-value
IL-1β*	Median (range)	0.18 (0.07–0.48)	0.26 (0.14–0.54)	**0.008**
	Mean ± SD	0.21 ± 0.10	0.31 ± 0.15	
IL-1ra*	Median (range)	20.75 (12.1–808.94)	28.87 (15.65–212.58)	0.074
	Mean ± SD	77.31 ± 172.76	41.68 ± 43.44	
IL-2^†^	Median (range)	0.69 (0.27–1.99)	1.01 (0.31–2.71)	**0.023**
	Mean ± SD	0.78 ± 0.38	1.26 ± 0.83	
IL-4^†^	Median (range)	0.50 (0.27–0.90)	0.67 (0.31–1.05)	**0.030**
	Mean ± *SD*	0.51 ± 0.16	0.65 ± 0.25	
IL-5*	Median (range)	0.00 (0.00–3.35)	0.41 (0.00–5.37)	0.144
	Mean ± *SD*	0.53 ± 0.89	1.28 ± 1.66	
IL-6*	Median (range)	3.45 (0.16–91.26)	7.25 (0.53–61.75)	0.253
	Mean ± *SD*	10.45 ± 17.27	12.17 ± 15.97	
IL-7*	Median (range)	19.84 (3.47–68.82)	14.82 (5.64–45.78)	0.197
	Mean ± *SD*	20.97 ± 13.87	16.37 ± 9.96	
IL-8*	Median (range)	63.63 (16.57–609.25)	83.26 (27.22–1758.28)	0.073
	Mean ± *SD*	107.89 ± 129.87	236.44 ± 395.22	
IL-9*	Median (range)	0.69 (0.00–2.02)	1.15 (0.34–3.76)	**0.020**
	Mean ± *SD*	0.81 ± 0.52	1.25 ± 0.79	
IL-10*	Median (range)	0.15 (0.00–7.14)	0.44 (0.03–2.99)	**0.002**
	Mean ± *SD*	0.45 ± 1.19	0.67 ± 0.69	
IL-12*	Median (range)	0.16 (0.00–0.68)	0.40 (0.00–0.73)	**0.011**
	Mean ± SD	0.22 ± 0.22	0.38 ± 0.22	
IL-13*	Median (range)	0.08 (0.00–0.72)	0.14 (0.00–1.07)	**0.031**
	Mean ± SD	0.12 ± 0.16	0.24 ± 0.28	
L-15^#^	Median (range)	–	–	–
	Mean ± *SD*	–	–	
IL-17A*	Median (range)	1.80 (0.74–3.21)	2.55 (1.34–3.36)	**0.008**
	Mean ± *SD*	1.82 ± 0.72	2.36 ± 0.67	
IL-18^†^	Median (range)	86.28 (13.95–169.54)	79.0 (0.00–124.68)	0.234
	Mean ± *SD*	86.21 ± 26.89	74.91 ± 33.52	
IFN-g*	Median (range)	39.01 (16.34–78.29)	43.40 (19.95–76.34)	0.261
	Mean ± *SD*	41.82 ± 16.25	47.25 ± 19.05	
TNF-a*	Median (range)	7.21 (2.96–18.09)	9.,54 (3.87–22.09)	**0.012**
	Mean ± *SD*	7.56 ± 3.14	10.39 ± 4.57	
CXCL12^†^	Median (range)	679.21 (222.29–1775.91)	763.48 (374.77–1329.40)	0.853
	Mean ± *SD*	756.17 ± 341.13	773.13 ± 277.77	
CXCL9*	Median (range)	518.69 (100.00–1536.56)	756.71 (100.00–4373.25)	**0.023**
	Mean ± *SD*	569.38 ± 361.70	1066.02 ± 1025.71	
MIF*	Median (range)	33818.30 (7570.28–191557.81)	39439.03 (22833.96–110441.88)	0.622
	Mean ± *SD*	56517.43 ± 46622.01	51879.01 ± 29619.94	
G-CSF*	Median (range)	6.85 (4.05–45.54)	8.36 (5.49–39.38)	**0.019**
	Mean ± *SD*	8.83 ± 7.39	11.17 ± 8.28	
GM-CSF*	Median (range)	0.00 (0.00–0.30)	0.00 (0.00–0.85)	0.082
	Mean ± *SD*	0.04 ± 0.08	0.14 ± 0.25	
M-CSF*	Median (range)	86.11 (0.00–535.72)	86.14 (0.00–400.00)	0.828
	Mean ± *SD*	107.14 ± 111.42	117.47 ± 117.74	
IP-10*	Median (range)	6244.22 (1944.4–30513.3)	8602.63 (1336.34–56177.70)	0.158
	Mean ± *SD*	8829.48 ± 6898.65	16163.94 ± 16871.34	
MCP-1^†^	Median (range)	615.58 (291.55–1226.51)	839.89 (406.26–1146.69)	**0.048**
	Mean ± *SD*	651.49 ± 210.79	766.83 ± 251.89	
MIP-1a*	Median (range)	1.37 (0.55–15.00)	1.63 (0.74–10.61)	0.283
	Mean ± *SD*	2.57 ± 3.48	2.75 ± 2.62	
MIP-1b*	Median (range)	6.76 (2.72–94.61)	6.83 (3.75–46.49)	0.749
	Mean ± *SD*	12.59 ± 17.88	11.80 ± 12.18	
RANTES*	Median (range)	3.75 (2.17–19.14)	4.73 (1.60–9.65)	**0.016**
	Mean ± *SD*	4.51 ± 2.90	5.32 ± 4.67	

*Kruskal–Wallis variance analysis (did not pass the Kolmogorov–Smirnov normality test), p-value < 0.05 was considered with statistical significance.

*^†^Independent t-test, p-value < 0.017 (0.05/3) was considered with statistical significance.*

*^#^IL-15 fell below the limit for all samples.*

*DME, diabetic edema; SD, standard deviation; IL, interleukin; IFN-g, interferon-g; TNF-a, tumor necrosis factor-a. CXCL, C-X-C chemokine ligand; MIF, macrophage-inhibiting factor; G-CSF, granulocyte-colony stimulating factor; GM-CSF, granulocyte-macrophage colony stimulating factor; M-CSF, macrophage colony stimulating factor; IP-10, interferon-inducible protein-10; MCP-1, monocyte chemoattractant protein-1; MIP, macrophage inflammatory protein; RANTES (CCL2), regulated upon activation normal T cell expressed and secreted. Bold values means with statistical significance.*

Further, we performed the correlation between the upregulated vitreous cytokines and the clinical characteristics of the included patients. We found that age was negatively correlated with the level of IL-1β (*p* = 0.05), IL-2 (*p* = 0.002), IL-9 (*p* = 0.034), TNF-α (*p* = 0.048), CXCL9 (*p* = 0.005), and RANTES (*p* = 0.024) while LogMAR BCVA was positively correlated with the level of IL-1β (*p* = 0.045), IL-9 (*p* = 0.018), IL-10 (*p* = 0.044), IL-12 (*p* = 0.003), IL-17A (*p* = 0.005), TNF-α (*p* = 0.02), CXCL9 (*p* = 0.024), and RANTES (*p* = 0.024) (Supplementary File 1).

## Discussion

Preoperative adjunctive anti-VEGF injection has been effectively used to decrease intraoperative bleeding, allowing for better visualization and more thorough fibrovascular membrane removal with fewer iatrogenic breaks (Stitt et al., 2016; Chelala et al., 2018). From the view of intraocular cytokines, we demonstrated a rapid decrease of VEGF-A after IVC but with no positive profibrotic switch within 7 days (Hu et al., 2019, 2021). In the present study, we further showed that preoperative IVC seemed not much influenced the expression of most inflammatory cytokines and chemokines, but the higher expression of which, might be associated with early postoperative ME.

Several anti-VEGF agents were available on the market, including bevacizumab, ranibizumab, aflibercept, conbercept, and brolucizumab. Bevacizumab, as a full-length (Fab and Fc) humanized murine IgG1 monoclonal antibody against VEGF-A, while Ranibizumab is a recombinant, humanized murine IgG1 monoclonal antibody fragment (Fab) targeting VEGF-A with higher affinity; Aflibercept is a recombinant fusion protein with VEGF receptors-1 and −2 fused to the Fc fragment of human IgG1; Similarly, conbercept is a recombinant fusion protein with high affinity for VEGF-A isoforms and PIGF; Brolucizumab is a single-chain antibody fragment targeting all forms of VEGF-A with higher solubility and lower molecular weight, allowing for administration of increased molar equivalents and in turn, may allow for longer intervals between doses. Though PDR and DME are two complications of diabetes mellitus and accumulating clinical and laboratory studies have evidenced the role of inflammation in the two complications, especially in DME (Hillier et al., 2017, 2018; Figueras-Roca et al., 2021; Hu et al., 2021; Mao et al., 2021; Minaker et al., 2021). However, studies assessing how the inflammatory cytokines and cytokines change after anti-VEGF therapy of DME have shown inconsistent findings. Some found that IV of anti-VEGF drugs had no influence on cytokines, while others detected statistically significant reduction of some cytokines, which may contribute to the anti-VEGF effect. Wei et al. (2019) showed that IVC did not cause significant differences in any inflammatory cytokines or growth factors in DME patients after 1 month. Sohn et al. (2011) also showed no difference of IL-6, IP-10, MCP-1, PDGF-AA in the IV of bevacizumab (IVB) group, but they were significantly decreased in the IV of triamcinolone acetonide (IVTA) group. Similarly, they demonstrated the findings in BRVO 4 weeks after IVB or IVTA (Sohn et al., 2014). On the other hand, Lim et al. (2018) showed there was a statistically significant reduction in VEGF, as well as IL-1β, IL-7, IL-8, IL-10, IL-12, IL-17, MCP-1, and TNF-α following two consecutive ranibizumab injections (IVR). Another two study also indicated IV-antiVEGF drugs lowered levels of aqueous VEGF-A and some inflammatory cytokines (Mastropasqua et al., 2018; Coughlin et al., 2020). In the study of Utsumi et al. (2021), however, there were decreased aqueous humor levels of VEGF, PlGF, PDGF-AA, and IP-10 but unchanged IL-6 and IL-8 1 month after IVR. Of note, two groups found that there were upregulated cytokines after anti-VEGF, such as GM-CSF (Mastropasqua et al., 2018) and IP-10 (Sato et al., 2018).

In comparison of the cytokines-associated studies mentioned above, the present study for the first time focused on earlier changes of cytokines after IVC in PDR patients (within 7 days after IVC). Among the 28 inflammatory cytokines and chemokines we measured, most did not alter remarkably, which was in line with the previously studies (Sohn et al., 2011; Wei et al., 2019). Such cytokines as IL-1β, TNF-α, and IL-12 are classical pro-inflammatory cytokines that might be secreted by microglia (M1) when cell surface receptors detect damage-associated molecular patterns (DAMPs) (Kinuthia et al., 2020). Alternatively, microglia can also shift to an anti-inflammatory phenotype (M2) and secrete anti-inflammatory cytokines including IL-4 and IL-10, and IL-13 (Cherry et al., 2014), which indicated that multiple microglia activation states in diabetic retinas. Either activated states microglia in retina requires chemokines, such as MCP-1, RANTES (CCL5), and CXCL9 to orchestrate microglia- neurons (or endothelia) action to modulate neuroprotective processes or limit retinal damage (Kinuthia et al., 2020). IL-17A, a proinflammatory cytokine mainly produced by T lymphocytes, has been recently demonstrated actively involved in DR pathophysiology by our group (Qiu et al., 2016, Qiu et al., 2021).

Our findings can be explained by that the IVC is selective to VEGF but not influential on inflammatory cytokines and chemokines, or the cytokines has not responded owing to the short period after IVC. The findings can also explain why there was no difference in occurrence of early ME between Pre-IV or No-Pre-IV group in the present study. Interestingly, we found TNF-α and IP-10 were upregulated after IVC, which has also been reported in the study of Juncal et al. (2020). We supposed that the upregulation of TNF-α and IP-10 might contribute to the injection manipulation (Agrawal et al., 2013).

Of note, despite the PPV procedure had cleared the VEGF and intravitreous cytokines, the baseline inflammatory cytokines profiles were still strongly associated with the early ME after surgery. Here, we detected a number of increased inflammatory cytokines and chemokines in patients with early ME in spite of antiVEGF or not. This indicated that after IVC and PPV surgery, there was still inflammatory status within the retinas. We believe this observation was of great clinical relevance, which further supported that anti-inflammation treatment at the end of, and after PPV surgery is important. Corticosteroids have been traditionally used to alleviate inflammation because of their ability to reduce leukocyte migration and cytokine production. Three potent synthetic corticosteroids, that is, triamcinolone acetonide (TA), dexamethasone and fluocinolone acetonide, was investigated as a treatment for diabetic macular edema (DME). Takamura et al. (2018) demonstrated that IVTA combined with vitrectomy contributed to reduce the anterior flare intensity and the early stage of DME in patients with VH due to PDR. In proliferative vitreoretinopathy, slow-release dexamethasone implant has also been suggested to reduce the ME after surgery (Banerjee et al., 2017).

This study also has several limitations. First, we had a limited sample size and the patients included had inconsistent PDR complex. Second, we could not evaluate the DME which already existed at the time of IVC because of the VH. Third, we did not evaluate the sham injection in a randomized study, which might influence the results. Finally, we did not investigate the long-term prognosis of ME or the correlation between the upregulated cytokines and the anti-inflammation therapy for those patients with early ME.

## Conclusion

In conclusion, the present study highlighted that the preoperative adjunctive Conbercept has limited influence on the vitreous inflammatory cytokines and chemokines in PDR. However, if the inflammatory cytokines and chemokines were elevated, they might be associated with early inflammation after vitrectomy, which indicated the importance of perioperative anti-inflammation. This study also provided valuable data and evidence for surgeons for developing new inflammatory anti-bodies for years to come. Future studies with more patients, more standard randomized control trial, different anti-VEGF agents, and longer follow-up are needed to further verify our conclusions.

## Data Availability Statement

The original contributions presented in the study are included in the article/Supplementary Material, further inquiries can be directed to the corresponding authors.

## Ethics Statement

This clinical trial was previously described and the clinical trial was registered at https://clinicaltrials.gov/ (ID NCT03506750). This study adhered to the tenets of the Declaration of Helsinki, and was approved by Ethic Committee of First Affiliated Hospital of Nanjing Medical University (2017-SR-283). The patients/participants provided their written informed consent to participate in this study. Written informed consent was obtained from the individual(s) for the publication of any potentially identifiable images or data included in this article.

## Author Contributions

HS and WZ analyzed the data and drafted the manuscript. ZH and QL designed to generated the conception, interpreted the data, and revised the manuscript. DH, ZZ, and JZ collected data. BQ, PX, and AM involved in discussion and review of the manuscript. All authors contributed to the article and approved the submitted version.

## Conflict of Interest

The authors declare that the research was conducted in the absence of any commercial or financial relationships that could be construed as a potential conflict of interest.

## Publisher’s Note

All claims expressed in this article are solely those of the authors and do not necessarily represent those of their affiliated organizations, or those of the publisher, the editors and the reviewers. Any product that may be evaluated in this article, or claim that may be made by its manufacturer, is not guaranteed or endorsed by the publisher.
