# Supplementary material for: Vitreous Inflammatory Cytokines and Chemokines, Not Altered After Preoperative Adjunctive Conbercept Injection, but Associated With Early Postoperative Macular Edema in Patients With Proliferative Diabetic Retinopathy
Source: Front Physiol. 2022 Mar 3;13:846003. doi: 10.3389/fphys.2022.846003 (PMC8928061; doi:10.3389/fphys.2022.846003)
Supplement: Supplementary file 1 [file Data_Sheet_1.DOCX]

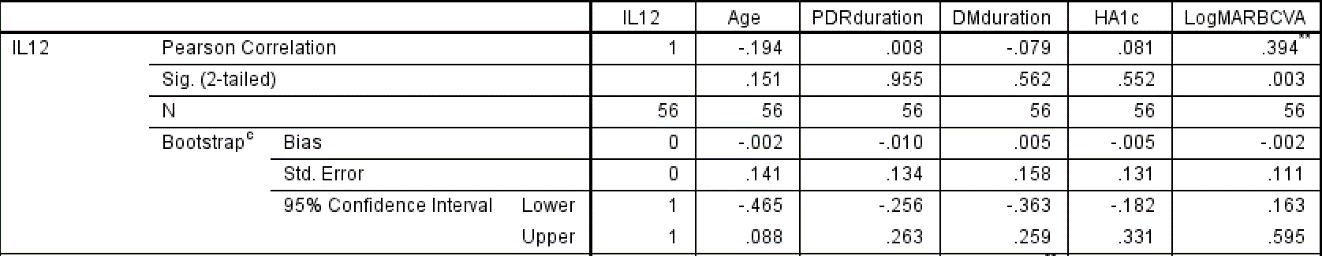

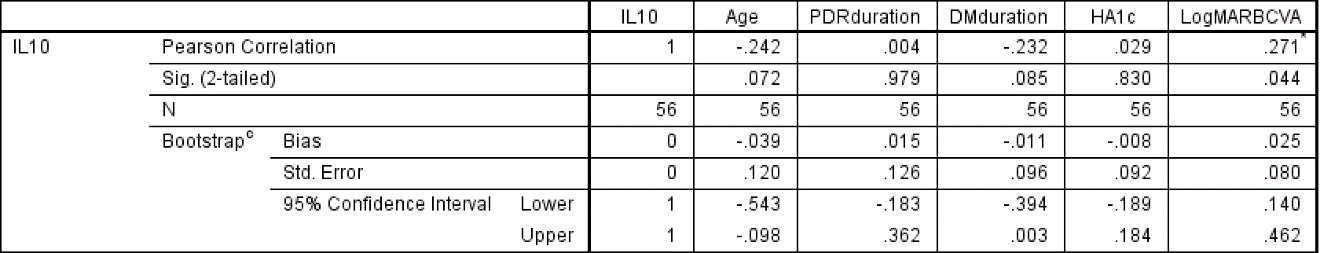

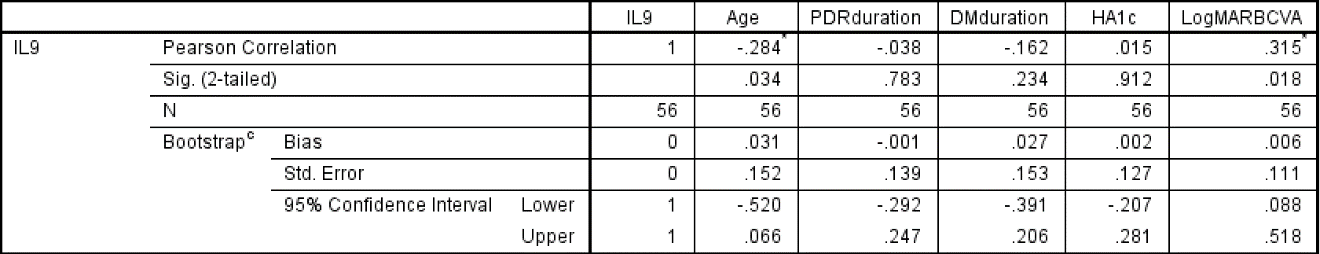

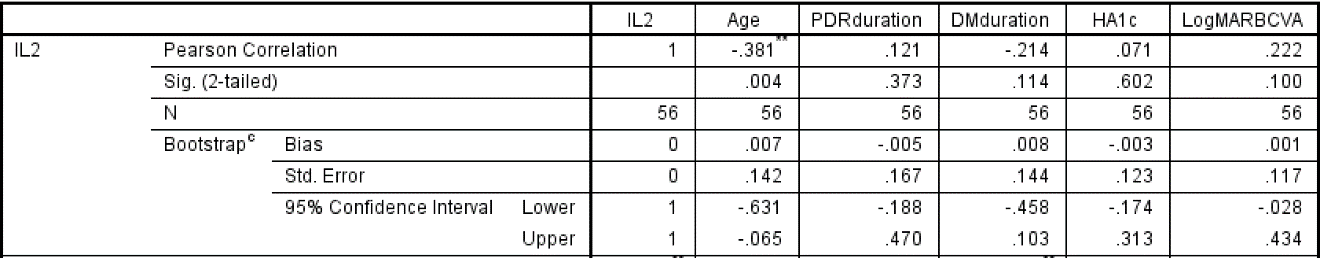

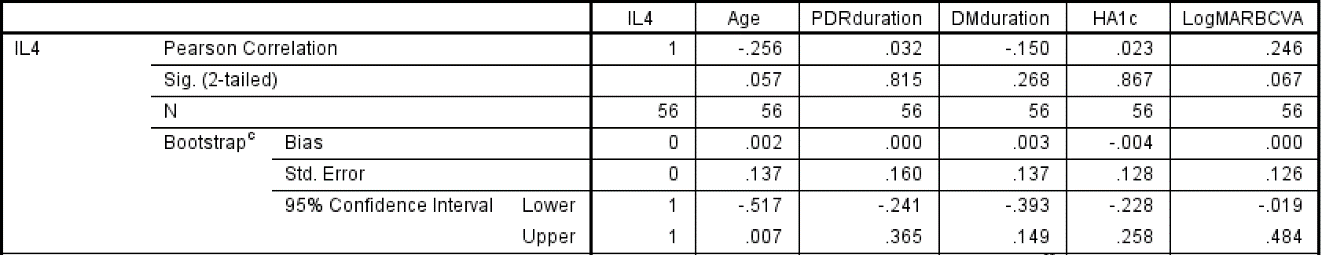

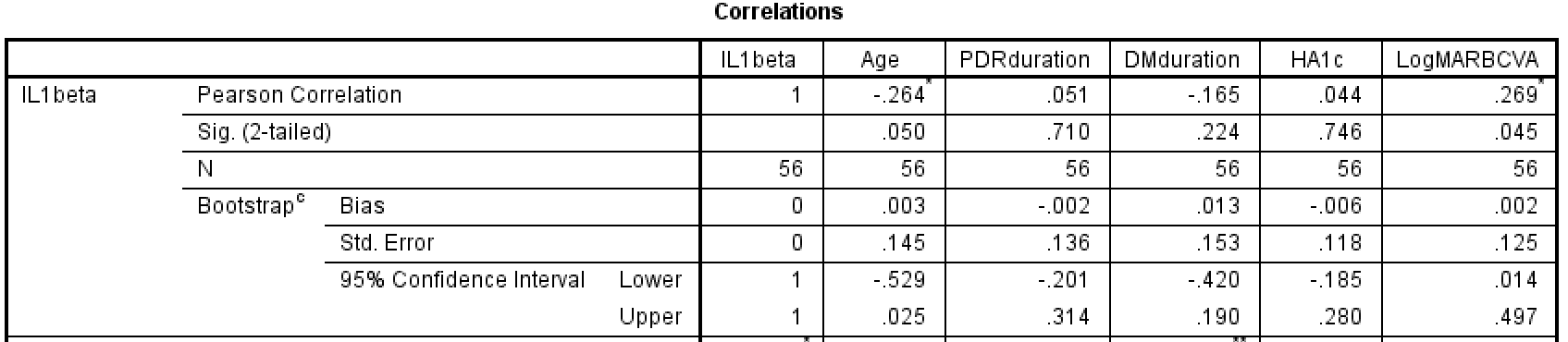


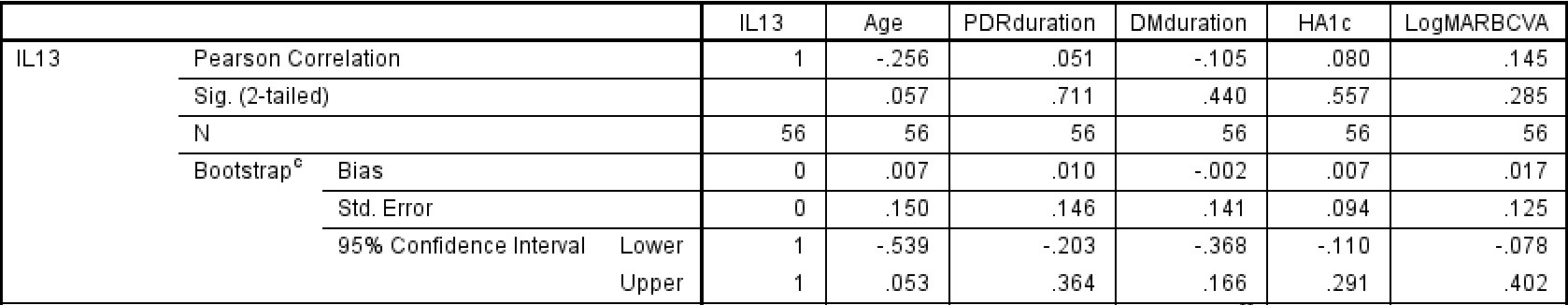


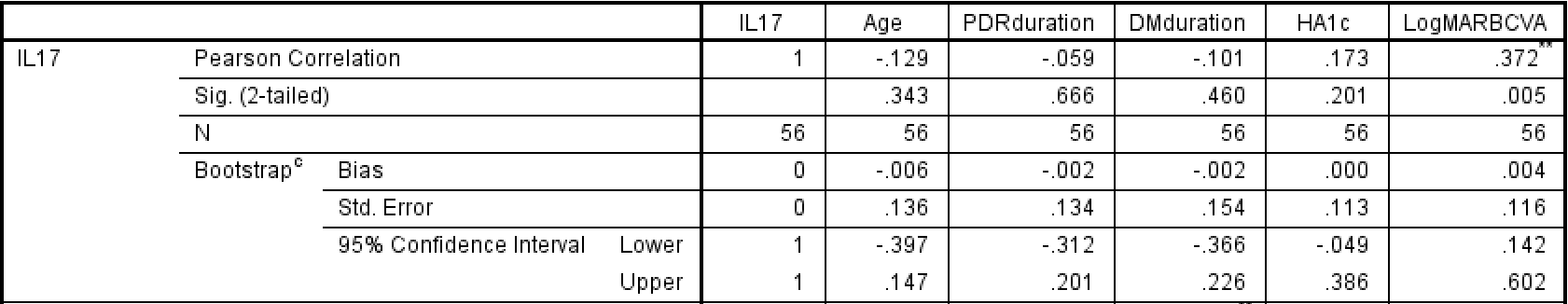


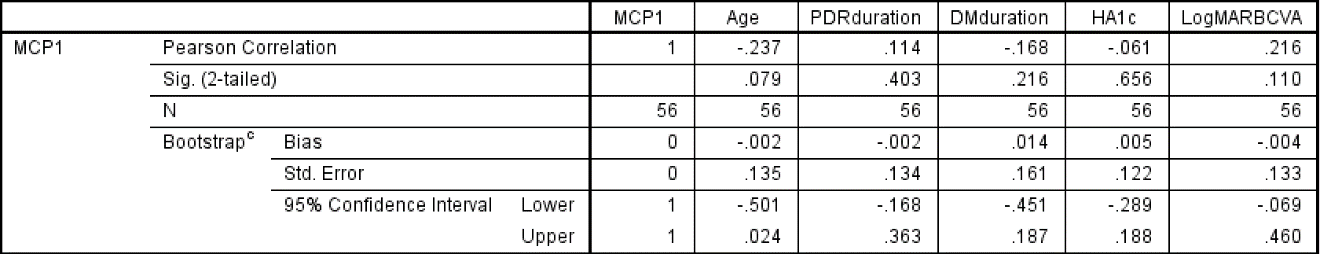

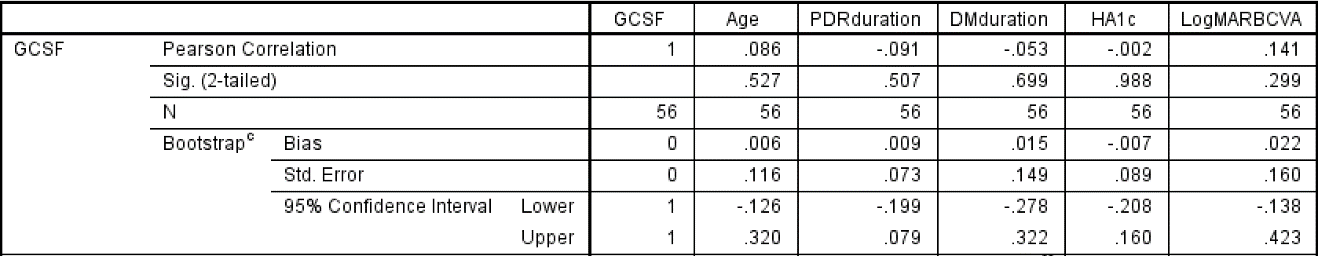

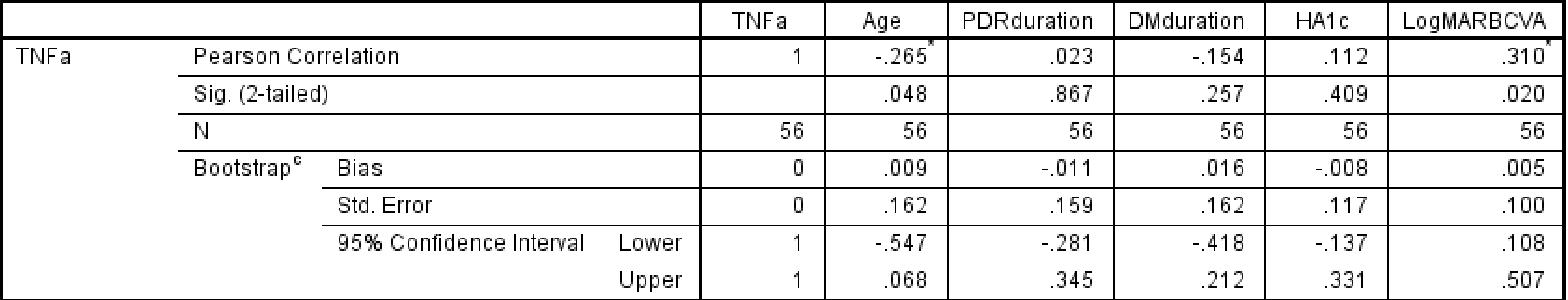


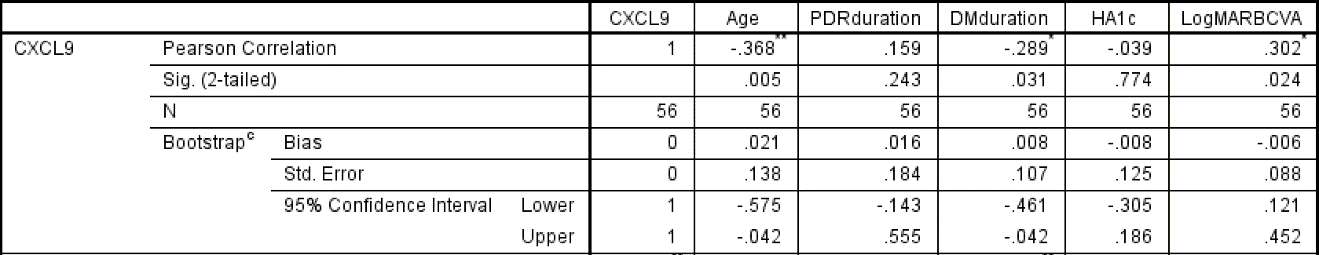


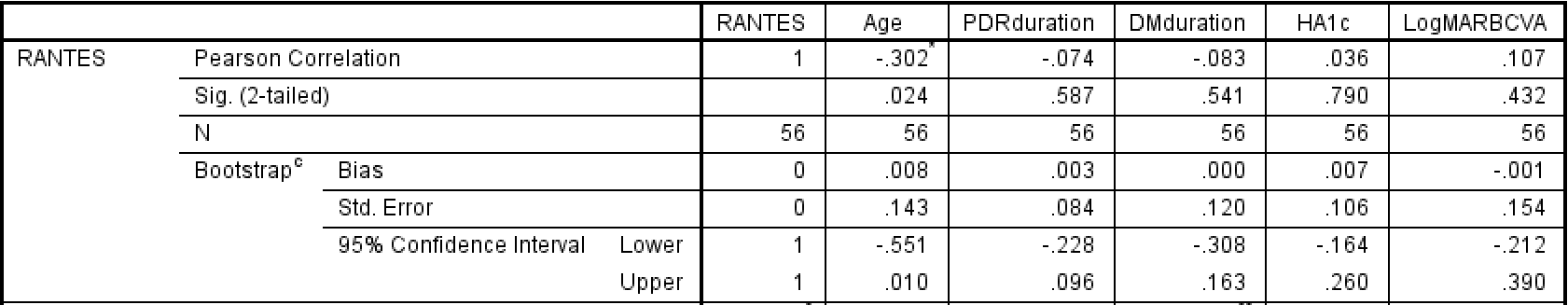


**Supplementary file 1.** Correlation between the upregulated vitreous cytokines and the clinical characteristics. * With statistical significance.
